# Heart Rate Variability Reactivity to Food Image Stimuli is Associated with Body Mass Index

**DOI:** 10.1007/s10484-021-09514-2

**Published:** 2021-05-22

**Authors:** Jung-Chi Chang, Wei-Lieh Huang, Chao-Yu Liu, Meg Mei-Chih Tseng, Cheryl C. H. Yang, Terry B. J. Kuo

**Affiliations:** 1 Department of Psychiatry, National Taiwan University Hospital, Taipei, Taiwan; 2 Department of Psychiatry, National Taiwan University Hospital Yunlin Branch, Yunlin, Taiwan; 3 Department of Psychiatry, College of Medicine, National Taiwan University, Taipei, Taiwan; 4 Graduate Institute of Clinical Medicine, National Taiwan University, Taipei, Taiwan; 5 Department of Clinical, Educational and Health Psychology, University College London, London, UK; 6 Department of Psychiatry, Far Eastern Memorial Hospital, New Taipei City, Taiwan; 7 Institute of Brain Science, National Yang-Ming University, Taipei, Taiwan

**Keywords:** Body mass index, Heart rate variability, Reactivity, Food

## Abstract

Appetitive control is driven by the hedonic response to food and affected by several factors. Heart rate variability (HRV) signals have been used to index autonomic activity and arousal levels towards visual stimuli. The current research aimed to examine the influence of body mass index (BMI), disordered eating behaviors, and sex on the HRV reactivity to food in a nonclinical sample. Thirty-eight healthy male and sixty-one healthy female participants completed questionnaires assessing disordered eating symptoms. HRV was recorded when the participants received visual stimuli of high-calorie food, neutral and negative emotional signals. Generalized estimating equation models were used to investigate the associations between HRV, BMI, disordered eating behaviors, and sex across the three stimulus types. Male participants demonstrated a higher ratio of low-frequency power to high-frequency power (LF/HF) than females across all the stimulus types. An increase in LF/HF reactivity to food signals was observed in all the study subjects. The moderation effect of BMI on LF/HF in response to food signals was also observed. Our study suggests that body weight may play a role in the interaction between sympathetic activity and food stimuli; however, how the interaction between sympathetic activity and food stimuli contributes to diet control warrants further investigation.

## Introduction

The prevalence of obesity has become a major public health concern over the last decades. The “obesity epidemic” imposes a substantial burden on the healthcare system as medical expenditures related to obesity and associated comorbidities are significantly higher than the normal weight population (Withrow & Alter, 2011). Overeating behavior is one of the causes of excessive energy supply and overweight (van Meer et al., 2016). Eating behavior per se is regulated by physical homeostasis and steered by the hedonic/reward value of food and appetitive control (Loeber et al., 2012). Higher cued-elicited responsivity to food not only predicts future weight gain but also links to binge eating behaviors in females (Stice et al., 2010). Individuals with obesity presented a heightened response to food in brain areas related to reward and diminished self-control capacity (van Meer et al., 2016). More importantly, the aforementioned psychophysiological responses to food can be detected early in the preclinical stage (Green et al., 2009).

It has been proposed that cue-elicited heart rate variability (HRV), a measurable psychophysiological response, may be associated with disordered eating behaviors (Friederich et al., 2006; Green et al., 2009). There are several common indices in HRV: (1) high-frequency power (HF) in HRV or root mean square successive differences (RMSSD) are usually viewed as proxies of heightened vagal tone (parasympathetic response); (2) low-frequency power (LF) of HRV reflects both sympathetic and parasympathetic influences (Laborde et al., 2017); (3) LF to HF ratio (LF/HF) can be taken as a proxy of sympatho-vagal balance under resting conditions (Fukunishi et al., 1999; Yamamoto et al., 1991) and (4) standard deviation of normal to normal RR interval (SDNN) or total power present overall variability with coverage of LF and HF (TASKEFORCE, 1996). Previous research has investigated the association between appetite, body weight, and different HRV indices (Green et al., 2009; Segerstrom & Nes, 2007; Udo et al., 2014). For example, it was found that exposure to palatable food increased LF (i.e., a proxy of sympatho-vagal influences) but exerted no noticeable influences on a proxy of vagal activity in normal-weight females (Nederkoorn et al., 2000). However, increased HF (i.e., a proxy of elevated parasympathetic response) was identified in individuals with obesity when exposed to visual food signals (Udo et al., 2014). One research study indicated that vagal activity (measured as the RMSSD) increased during individuals’ resistance to high-caloric food (Segerstrom & Nes, 2007). It is also noteworthy that HRV reactivity may be subject to other factors. Obesity has been found to affect cardiac autonomic control hence HRV reactivity (Paschoal et al., 2009; Vanderlei et al., 2010). Other factors such as disordered eating behaviors and different experimental conditions may also contribute to the mixed results. Nevertheless, previous literature still supports cue-elicited HRV as a useful psychophysiological marker of ineffective appetitive regulation, disordered eating behaviors, and risk of future weight gain (Green et al., 2009; Segerstrom & Nes, 2007).

Despite the knowledge that heightened psychophysiological sensitivity toward food-related stimuli can predict disordered eating and weight gain, little research has been performed to investigate the complex association between disordered eating behaviors, BMI, sex on the psychophysiological response to food. The current study aimed to examine the interrelatedness of disordered eating behaviors, BMI, and physiological responses to high-calorie food. We hypothesized that the BMI values of the subjects and disordered eating behaviors can be associated with their HRV reactivity to food signals.

## Materials and Methods

### Participants and Procedure

We recruited participants from the National Taiwan University Hospital (NTUH) Yunlin Branch in 2015. This study was approved by the Research Ethics Committee of NTUH (approval Id: 201412025RINB). Subjects aged between 20 and 65 years were recruited through posters and internet advertisements. Subjects were screened, and those with mental retardation, psychotic disorders, substance use disorders, and cardiovascular diseases such as hypertension and coronary artery disease were excluded. Written informed consent was obtained from all the participants before inclusion.

**Fig. 1:**
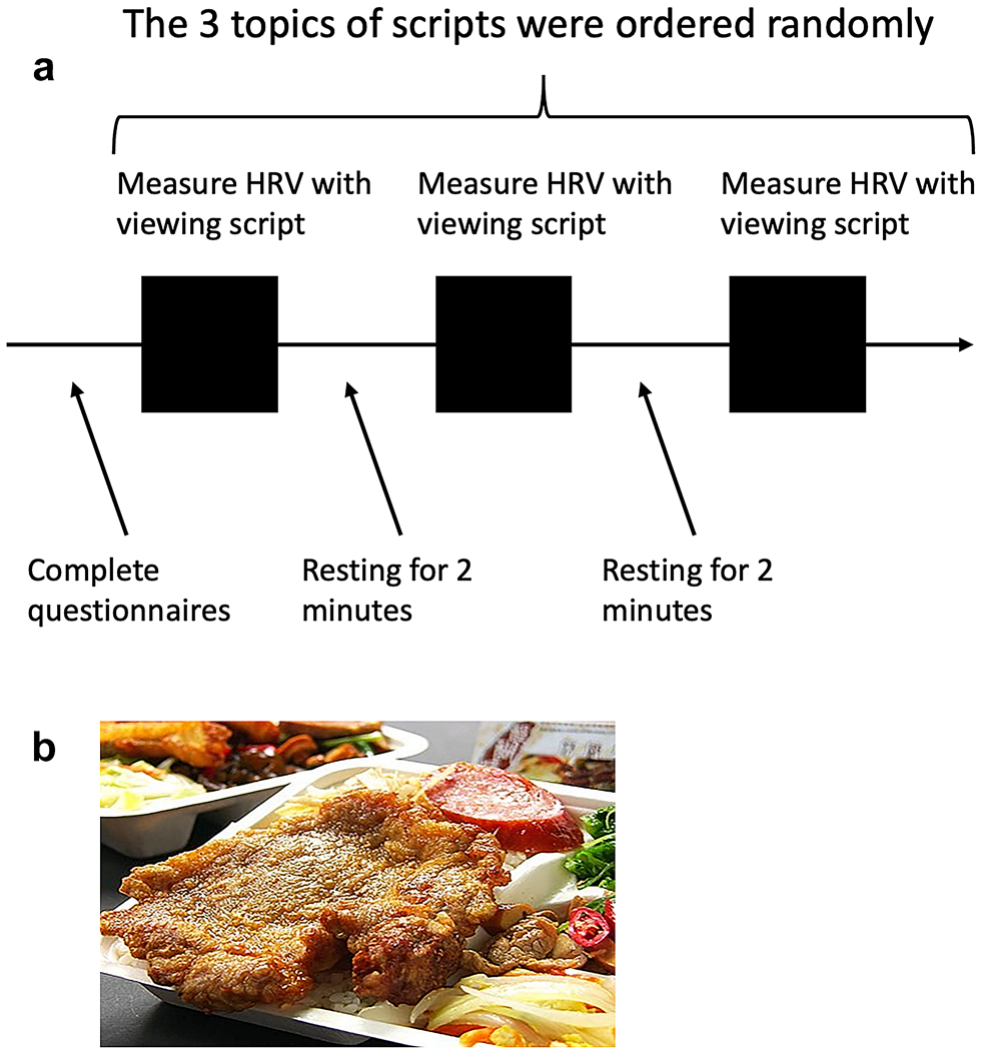
**a** Schematic of the experimental procedure. **b** Illustration of food stimulus, presented as food pictures displayed on a 16' screen over a white background. *HRV* heart rate variability

The schematic procedure of this study is illustrated in Fig. 1, based on the recommendation of a previous HRV study (Laborde et al., 2017). Following initial assessments, each participant was instructed to sit comfortably and watch films presented on a 17-inch computer screen while heart rate was recorded. Videos are 5-min long with three different themes (neutral stimuli, high-calorie savory food stimuli, and negative emotional stimuli) in random order. Neutral stimuli included 23 images of household objects and street scenes. Food stimuli included 23 pictures of high-calorie food or drinks, such as cakes, hamburgers, and pizza. Negative emotion stimuli included 23 photos showing people in agony or tragic scenes. Three films were presented in a random sequence with a 2-min interval in between.

### Psychological Measurements

In this study, disordered eating behavior was evaluated by the Bulimic Investigatory Test, Edinburgh (BITE) and the eating disorder examination questionnaire (EDEQ). BITE, a brief self-reported questionnaire, consists of the Symptom subscale (30 items) and the Severity subscale (6 items) to examine bulimic symptomatology. The Symptom scale provides a dichotomous response format, while responses to the Severity scale are scored with the frequency of bingeing or purging (Henderson & Freeman, 1987). Good internal consistency (Cronbach alpha 0.95 and 0.77 for the Symptom and Severity scales) and test-retest reliability (0.86 and 0.88 for the Symptom and Severity scales) have been demonstrated for the Chinese version of the BITE (Tseng et al., 2004).

EDEQ is a self-reported measure that assesses behaviors and attitudes related to eating disorders in the past 28 days (Fairburn & Beglin, 1994). Studies showed this self-report questionnaire bears a high level of agreement with its original version (Carter et al., 2001). Good test-retest reliability (0.70–0.93) and internal consistency (Cronbach’s alpha = 0.72–0.94) of the Chinese version of EDEQ were reported (Tu et al., 2017).

### Heart Rate Variability

An HRV analyzer (SS1C, Enjoy Research Inc., Taiwan) was used to record the electrocardiogram (ECG) for 5 min continuously with a standard procedure (TASKEFORCE, 1996). The participants were asked not to fall asleep, sit quietly and breathe normally. An 8-bit analog-to-digital converter was used to sampled signals at 512 Hz. We used the HRV analyzer to analyze and process the digitized ECG signals and also store on a hard disk simultaneously. The computer calculation was used to inspected each QRS complexes and the unordinary signals, for example, noise or premature ventricular constrictions were dismissed by the probability of fitting a standard template. Normal and stationary R-R interval values were detected and interpolated at a rate of 7.11 Hz to produce continuity in the time domain. For frequency domain analysis of the R-R wave intervals, LF was calculated within the frequency range of 0.04–0.15 Hz, and HF was calculated within the frequency range of 0.15–0.4 Hz (Berntson et al., 1997). LF/HF ratio was also calculated. SDNN (a total variability indicator), HF (a specific parasympathetic indicator), and LF/HF (a sympathetic or balance indicator) were chosen as the main HRV indicators in current research. More details of the algorithm can be found in the previous literature (Huang et al., 2012, 2017).

### Statistical Analysis

Descriptive statistics were used for estimating demographics, scores of questionnaires, and HRV data. We used generalized estimating equation analysis (GEE) to examine the impact of the demographic and psychological variables on HRV. An exchangeable working correlation structure and the robust standard error estimator were applied in our analyses (Liang & Zeger, 1986). SDNN, HF, and LF/HF were set as dependent variables, whereas independent variables included stimuli, demographics (sex, age, BMI), disordered eating behaviors (BITE scores and EDEQ scores), and interaction items (stimuli ^*^BMI/BITE scores/EDEQ scores). All alpha values were set as 0.05, and two-sided analyses were used. The statistical analysis was conducted with SPSS 25 (IBM, USA).

## Results

Ninety-nine eligible participants completed the study (38 males and 61 females). The mean age of all the participants was 28.77 years old, and the mean BMI was 23.06 kg/m^2^. The mean score of BITE is 6.26, and the mean score of EDEQ is 9.06. The demographics, eating features, and HRV of all the participants are presented in Table 1.

**Table 1:** Demographic, clinical characteristics, and HRV of all participants

	Total (N = 99)
	Mean ± SD
Age (yrs)	28.77 ± 6.04
Sex (male) (n,%)	38, 38.38%
BMI (kg/m2)	23.06 ± 4.59
BITE	6.26 ± 4.14
EDEQ	9.06 ± 6.97
SDNN (ms)	
During negative emotion stimuli	42.97 ± 16.39
During food stimuli	43.31 ± 17.26
During neutral stimuli	41.76 ± 16.63
HF [ln(ms^2^)]	
During negative emotion stimuli	5.88 ± 0.98
During food stimuli	5.88 ± 1.03
During neutral stimuli	5.82 ± 1.03
LF/HF [ln(ratio)]	
During negative emotion stimuli	0.05 ± 0.86
During food stimuli	0.00 ± 0.85
During neutral stimuli	0.04 ± 0.82

*HRV* heart rate variability, *SDNN* standard deviation of normal to normal, *HF* high-frequency power, *LF/HF* ratio of low-frequency power to high-frequency power, *BMI* body mass index, *BITE* Bulimic Investigatory Test, Edinburgh, *EDEQ* Eating Disorder Examination Questionnaire

The GEE models of HRV of all the participants measured during different stimuli exposure are summarized in Table 2. The level of LF/HF had a positive correlation with age, and male participants presented with higher in LF/HF than females during all stimuli. When watching the high-calorie food video, all participants presented with significantly higher LF/HF than during the neutral. There is no significant difference in LF/HF during negative emotion induction compared to neutral stimuli. Furthermore, a significant negative correlation was found between the score of EDEQ and LF/HF and a positive correlation between the score of BITE and LF/HF but without reaching a significant level.

**Table 2:** The biological and psychological effects on heart rate variability during neutral, food, and negative emotion stimuli

Outcomes	SDNN (ms)		HF [ln(ms^2^)]		LF/HF [ln(ratio)]	
Variables	Estimates (S.E.)	p value	Estimates (S.E.)	p value	Estimates (S.E.)	p value
Visual Stimuli						
During negative emotion stimuli (NE)	− 2.64 (3.54)	0.456	− 0.16 (0.21)	0.458	0.20 (0.33)	0.547
During food stimuli (F)	1.46 (3.42)	0.670	− 0.15 (0.17)	0.386	0.61 (0.22)	0.007**
During neutral stimuli (NS, reference)	–	–	–	–	–	–
Demographics						
Female	− 6.24 (3.73)	0.094	− 0.08 (0.23)	0.734	− 0.48 (0.18)	0.007**
Male (reference)	–	–	–	–	–	–
Age	− 0.86 (0.28)	0.002*	− 0.04 (0.02)	0.011*	0.03 (0.01)	0.008**
BMI	− 0.33 (0.53)	0.537	− 0.06 (0.04)	0.128	0.01 (0.02)	0.399
Eating features						
BITE	− 0.56 (0.43)	0.192	− 0.02 (0.02)	0.295	0.03 (0.02)	0.060
EDEQ	0.37 (0.32)	0.240	0.03 (0.02)	0.096	− 0.02 (0.01)	0.021*
Interaction items						
BMI × NE	0.16 (0.12)	0.196	0.01 (0.01)	0.380	− 0.01 (0.01)	0.484
BMI × F	− 0.02 (0.13)	0.872	0.01 (0.01)	0.204	− 0.03 (0.01)	0.001**
BMI × NS (reference)	–	–	–	–	–	–
BITE × NE	− 0.30 (0.22)	0.174	0.00 (0.01)	0.953	− 0.01 (0.01)	0.521
BITE × F	− 0.07 (0.20)	0.739	− 0.01 (0.01)	0.616	0.00 (0.01)	0.982
BITE × NS (reference)	–	–	–	–	–	–
EDEQ × NE	0.23 (0.13)	0.080	0.00 (0.01)	0.554	0.01 (0.01)	0.253
EDEQ × F	0.11 (0.13)	0.376	0.00 (0.01)	0.706	0.00 (0.01)	0.640
EDEQ × NS (reference)	–	–	–	–	–	–

*SDNN* standard deviation of normal to normal, *HF* high-frequency power, *LF/HF* ratio of low-frequency power to high-frequency power, *BMI* body mass index, *BITE* Bulimic Investigatory Test, Edinburgh, *EDEQ* Eating Disorder Examination Questionnaire

*p < 0.05

**p < 0.01

***p < 0.001

We also examined the effects of interaction between stimuli and other factors on HRV. There was a BMI^*^ food stimuli interaction to LF/HF (p = 0.001). That is, when watching the high-calorie video, we found the participants with higher BMI were less sensitive toward visual food stimuli in LF/HF compared to lower BMI people. This phenomenon was not observed in the interaction between BMI^*^ negative emotion induction relative to neutral stimuli. Besides, no significant results were found about the interaction between disordered eating behaviors and stimuli. We also applied a similar GEE model to HF and SDNN; only significant positive associations between HF-age and SDNN-age were observed; these two HRV indicators were not significantly associated with stimuli.

## Discussion

Our study found increased LF/HF reactivity under high-calorie food stimuli compared to neutral stimuli in all the study subjects. This is consistent with the study that nonclinical subjects react to food stimuli with increased LF. The increased LF/HF under food stimuli may suggest mental efforts or cognitive processes (Nederkoorn et al., 2000). Moreover, the current report also found a negative association between higher BMI values and LF/HF reactivity under food stimuli. Previous studies showed that LF/HF could be used as a proxy of sympathetic and parasympathetic coordination (Berntson et al., 1997). It can also reflect neurophysiological processes, including cognitive restraint (Takada et al., 2018). Cognitive restraint of food intake represents the conscious mechanism of restraining oneself from eating to control body weight and can be investigated with the Three-Factor Eating Questionnaire by AJ Stunkard and S Messick (Cappelleri et al., 2009; Stunkard & Messick, 1985). Our findings may thus imply that weight status can moderate the relation between autonomic response and cognitive restraint of food intake. As a result, individuals with higher BMI exhibit more dysfunction in sympathetic and parasympathetic synchronization and weaker cognitive restraint when encountered with high-calorie food, leading to more food intake. Previous studies mainly focused on the involvement of parasympathetic functioning in the development of obesity (Laederach-Hofmann et al., 2000; Spitoni et al., 2017; Udo et al., 2014). The role of sympathetic functioning in appetitive control was less clear. More research is needed to explore whether the combination of sympathetic and parasympathetic activity during food stimuli is associated with diet control.

Our results are in accordance with previous studies showing that males demonstrated higher LF/HF across different stimulus types (Bigger et al., 1995; Kuo et al., 1999; Park et al., 2007; Voss et al., 2013). For example, Park et al. (2007) found that men presented higher LF/HF than women in a large sample of healthy participants in the resting condition. These findings demonstrated sex differences in autonomic functioning in heart rate control. Men aged 40–59 years exhibited greater %LF and LF/HF ratios compared to women, and women aged 40–59 years had greater HF powers compared to men (Kuo et al., 1999).

The current study showed that LF/HF was negatively correlated with symptom severity of disordered eating behaviors. The result was consistent with previous research that autonomic dysfunction is associated with disordered eating behaviors (Green et al., 2009). However, no associations were found between food stimulus and HF or with SDNN. HF is considered to be the HRV indices with specific physiological significance of the parasympathetic activity. SDNN is also thought to be more associated with the activity of the parasympathetic system (TASKEFORCE, 1996). We suggested that the stimulus-oriented response involves more changes in sympathetic activity; which resulted in non-significant correlation between HF and food stimulus. Therefore, the indicators which can reflect more sympathetic activity, such as LF/HF (often considered to be the balance of sympathetic and parasympathetic activity) can be more sensitive to reflect this process finding. We proposed that the stimulus-oriented response involves more changes in sympathetic activity; which resulted in a non-significant correlation between HF and food stimulus. Therefore, the indices which reflect more sympathetic activity, such as LF/HF (often considered to be the balance of sympathetic and parasympathetic activity) may be more sensitive to detect such a response.

Besides, our results did not find the interaction between disordered eating behaviors and any visual stimuli when predicting the HRV response. It may be due to the relatively modest sample size and mild disordered eating behaviors among study subjects.

Our findings indicated that sympathetic reactivity would increase during food stimuli. In addition, an attenuated pattern in sympathetic reactivity was observed in participants with higher BMI. Image studies had shown that successful weight loss was correlated with a functional brain subnetwork of sensory regions (Levakov et al., 2021; Stice & Burger, 2019). Our results might provide evidence of the association between body weight and the sensitivity to food cues. Taken together, the current study provides evidence of the association between body weight and the sensitivity to food cues which helps to understand the etiology of obesity and the mechanism underlying dietary control.

This study has the following limitations. First, the study sample was composed of young and healthy individuals with an average BMI ranging from 21.92 to 24.90. Therefore, whether the results can be applied to the patient with the diagnosis of anorexia nervosa or bulimia nervosa needs further exploration. However, the significant association between BMI and HRV food reactivity in the nonclinical sample indicates an aberrant neurophysiologic response to food could be detected before disease onset, thus serving a possible risk factor of obesity. Second, the satiety state of each participant was not investigated. However, evidence indicates that subjective hunger and food intake do not influence HRV or other neurophysiological responses to food in normal subjects (Nederkoorn et al., 2000). Third, the traditional interpretation of HRV parameter has been challenged with regard to an over-simplified framework of HRV in the past (Hayano & Yuda, 2019). For example, Reyes et al. (2013) suggested that LF may be the proxy of the parasympathetic system, rather than the sympathetic system. Therefore, we should be more cautious when interpreting the physiological meanings of the HRV indices. Finally, the influences of physical activity and respiration were not controlled in our analysis. Nevertheless, previous data regarding the effect of breathing found satisfactory reproducibility of short-term HRV measurement without strict respiration control (Schipke et al., 1999). Also, physical activity levels did not seem to affect autonomic activity in young subjects (Gregoire et al., 1996). Therefore, the influences of respiration and physical activity on the findings may be negligible.

## Conclusion

Negative correlations were found between LF/HF and symptom severity of disordered eating behaviors as well as between BMI and sensitivity to food stimuli. Furthermore, BMI moderated the relationship between food stimuli and LF/HF, which is a proxy for the sympathy-vagal balance. Future work should thus examine how the interaction between sympathetic activity and food stimuli is related to weight gain over time in both clinical and nonclinical populations.

## References

[CR1] Berntson GG, Bigger JT, Eckberg DL, Grossman P, Kaufmann PG, Malik M, Nagaraja HN, Porges SW, Saul JP, Stone PH, van der Molen MW (1997). Heart rate variability: Origins, methods, and interpretive caveats. Psychophysiology.

[CR2] Bigger JT, Fleiss JL, Steinman RC, Rolnitzky LM, Schneider WJ, Stein PK (1995). RR variability in healthy, middle-aged persons compared with patients with chronic coronary heart disease or recent acute myocardial infarction. Circulation.

[CR3] Cappelleri JC, Bushmakin AG, Gerber RA, Leidy NK, Sexton CC, Karlsson J, Lowe MR (2009). Evaluating the power of food scale in obese subjects and a general sample of individuals: Development and measurement properties. International Journal of Obesity.

[CR4] Carter JC, Aime AA, Mills JS (2001). Assessment of bulimia nervosa: A comparison of interview and self-report questionnaire methods. International Journal of Eating Disorders.

[CR5] Fairburn CG, Beglin SJ (1994). Assessment of eating disorders: Interview or self-report questionnaire?. International Journal of Eating Disorders.

[CR6] Friederich HC, Schild S, Schellberg D, Quenter A, Bode C, Herzog W, Zipfel S (2006). Cardiac parasympathetic regulation in obese women with binge eating disorder. International Journal of Obesity.

[CR7] Fukunishi I, Sei H, Morita Y, Rahe RH (1999). Sympathetic activity in alexithymics with mother’s low care. Journal of Psychosomatic Research.

[CR8] Green MA, Hallengren JJ, Davids CM, Riopel CM, Skaggs AK (2009). An association between eating disorder behaviors and autonomic dysfunction in a nonclinical population. A pilot study. Appetite.

[CR9] Gregoire J, Tuck S, Yamamoto Y, Hughson RL (1996). Heart rate variability at rest and exercise: influence of age, gender, and physical training. Canadian Journal of Applied Physiology.

[CR10] Hayano J, Yuda E (2019). Pitfalls of assessment of autonomic function by heart rate variability. Journal of Physiological Anthropology.

[CR11] Henderson M, Freeman CP (1987). A self-rating scale for bulimia. The 'BITE'. British Journal of Psychiatry.

[CR12] Huang WL, Liao SC, Yang CC, Kuo TB, Chen TT, Chen IM, Gau SSF (2017). Measures of heart rate variability in individuals with somatic symptom disorder. Psychosomatic Medicine.

[CR13] Huang WL, Lin YH, Kuo TB, Chang LR, Chen YZ, Yang CC (2012). Methadone-mediated autonomic functioning of male patients with heroin dependence: The influence of borderline personality pattern. PLoS ONE.

[CR14] Kuo TB, Lin T, Yang CC, Li CL, Chen CF, Chou P (1999). Effect of aging on gender differences in neural control of heart rate. The American Journal of Physiology.

[CR15] Laborde S, Mosley E, Thayer JF (2017). Heart rate variability and cardiac vagal tone in psychophysiological research–recommendations for experiment planning, data analysis, and data reporting. Frontiers in Psychology.

[CR16] Laederach-Hofmann K, Mussgay L, Rúddel H (2000). Autonomic cardiovascular regulation in obesity. The Journal of Endocrinology.

[CR17] Levakov G, Kaplan A, Yaskolka Meir A, Rinott E, Tsaban G, Zelicha H, Meiran N, Shelef I, Shai I, Avidan G (2021). Neural correlates of future weight loss reveal a possible role for brain-gastric interactions. NeuroImage.

[CR18] Liang K-Y, Zeger SL (1986). Longitudinal data analysis using generalized linear models. Biometrika.

[CR19] Loeber S, Grosshans M, Korucuoglu O, Vollmert C, Vollstadt-Klein S, Schneider S, Wiers RW, Mann K, Kiefer F (2012). Impairment of inhibitory control in response to food-associated cues and attentional bias of obese participants and normal-weight controls. International Journal of Obesity.

[CR20] Nederkoorn C, Smulders FT, Jansen A (2000). Cephalic phase responses, craving and food intake in normal subjects. Appetite.

[CR21] Park SB, Lee BC, Jeong KS (2007). Standardized tests of heart rate variability for autonomic function tests in healthy Koreans. The International Journal of Neuroscience.

[CR22] Paschoal MA, Trevizan PF, Scodeler NF (2009). Heart rate variability, blood lipids and physical capacity of obese and non-obese children. Arquivos Brasileiros De Cardiologia.

[CR23] Reyes del Paso GA, Langewitz W, Mulder LJ, Van Roon A, Duschek S (2013). The utility of low frequency heart rate variability as an index of sympathetic cardiac tone: a review with emphasis on a reanalysis of previous studies. Psychophysiology.

[CR24] Schipke J, Arnold G, Pelzer M (1999). Effect of respiration rate on short-term heart rate variability. Journal of Clinical and Basic Cardiology.

[CR25] Segerstrom SC, Nes LS (2007). Heart rate variability reflects self-regulatory strength, effort, and fatigue. Psychological Science.

[CR26] Spitoni GF, Ottaviani C, Petta AM, Zingaretti P, Aragona M, Sarnicola A, Antonucci G (2017). Obesity is associated with lack of inhibitory control and impaired heart rate variability reactivity and recovery in response to food stimuli. International Journal of Psychophysiology.

[CR27] Stice E, Burger K (2019). Neural vulnerability factors for obesity. Clinical Psychology Review.

[CR28] Stice E, Yokum S, Bohon C, Marti N, Smolen A (2010). Reward circuitry responsivity to food predicts future increases in body mass: Moderating effects of DRD2 and DRD4. NeuroImage.

[CR29] Stunkard AJ, Messick S (1985). The three-factor eating questionnaire to measure dietary restraint, disinhibition and hunger. Journal of Psychosomatic Research.

[CR30] Takada K, Ishii A, Matsuo T, Nakamura C, Uji M, Yoshikawa T (2018). Neural activity induced by visual food stimuli presented out of awareness: A preliminary magnetoencephalography study. Science and Reports.

[CR31] TASKEFORCE (1996). Heart rate variability: Standards of measurement, physiological interpretation and clinical use. Task force of the European society of cardiology and the North American society of pacing and electrophysiology. Circulation.

[CR32] Tseng MC, Lee MB, Chen SY, Lee YJ, Lin KH, Chen PR, Lai JS (2004). Response of Taiwanese obese binge eaters to a hospital-based weight reduction program. Journal of Psychosomatic Research.

[CR33] Tu C-Y, Tseng M-C, Chang C-H, Lin C-C (2017). Comparative validity of the Internet and paper-and-pencil versions of the night eating questionnaire. Comprehensive Psychiatry.

[CR34] Udo T, Weinberger AH, Grilo CM, Brownell KD, DiLeone RJ, Lampert R, Matlin SL, Yanagisawa K, McKee SA (2014). Heightened vagal activity during high-calorie food presentation in obese compared with non-obese individuals–results of a pilot study. Obesity Research & Clinical Practice.

[CR35] van Meer F, Charbonnier L, Smeets PAM (2016). Food decision-making: Effects of weight status and age. Current Diabetes Reports.

[CR36] Vanderlei LCM, Pastre CM, Freitas IF, de Godoy MF (2010). Analysis of cardiac autonomic modulation in obese and eutrophic children. Clinics.

[CR37] Voss A, Schroeder R, Fischer C, Heitmann A, Peters A, Perz S (2013). Influence of age and gender on complexity measures for short term heart rate variability analysis in healthy subjects. Conference Proceedings: Annual International Conference of the IEEE Engineering in Medicine and Biology Society.

[CR38] Withrow D, Alter DA (2011). The economic burden of obesity worldwide: A systematic review of the direct costs of obesity. Obesity Reviews.

[CR39] Yamamoto Y, Hughson RL, Peterson JC (1991). Autonomic control of heart rate during exercise studied by heart rate variability spectral analysis. Journal of Applied Physiology (1985).

